# Financial incentives for women's cervical, breast and colorectal cancer screening adherence: systematic review and meta-analysis

**DOI:** 10.1016/j.pmedr.2026.103507

**Published:** 2026-05-25

**Authors:** Cristina Lumia, Antonella Nespoli, Davide Ausili, Simona Fumagalli, Alexandre Dumont

**Affiliations:** a University of Milano-Bicocca, School of Medicine and Surgery, Italy; b Centre Population & Développement (CEPED), Université de Paris Cité, France

**Keywords:** Screening, Financial incentives, Cancer prevention, Behavioural economics, Adherence, Women's health, Public health

## Abstract

**Objective:**

To evaluate the effectiveness of financial incentives on women's participation in cervical, breast, and colorectal cancer screening.

**Methods:**

We conducted a systematic review and meta-analysis of quantitative studies evaluating financial incentives to increase women's participation in cancer screening. MEDLINE, CINAHL, and the Cochrane Library were searched from December 2024 to June 2025. Two reviewers independently screened studies, extracted data, and assessed risk of bias. Certainty of evidence was assessed using GRADE. The review protocol was registered in PROSPERO (CRD42025640357).

**Results:**

Eleven studies were included. Financial incentives were associated with increased screening participation - RR 1.42 (95% CI 1.05, 1.92); I^2^ = 98.30%. Effect estimates varied across follow-up time windows, with larger point estimates observed when screening uptake was assessed within ≤1 month of incentive delivery – RR 2.69 (95% CI 1.05, 6.89) - and effects remained detectable at approximately one year - RR 1.57 (95% CI 1.00, 2.46). Incentives in the range of approximately USD 10–25 were commonly used across trials and were often associated with positive effects.

**Conclusions:**

Financial incentives may improve women's screening participation. Further studies are needed to evaluate the effectiveness of different incentive magnitudes and delivery strategies, alone or in combination with other interventions.

## Highlights

• Modest financial incentives may increase women's cancer screening adherence.
• Most studies evaluated incentives of about $10–25, limiting dose-response comparisons.
• Effects varied across follow-up periods, contexts, and target screening behaviors.
• Heterogeneity likely reflects differences in context, incentive design, and behavior.
• Further research should assess optimal design, long-term outcomes, and equity.

## 1 Introduction

Cancer represents a major public health burden among women and is currently the second leading cause of death among women in the United States (Kenealy and Lochner, 2025). Breast and colorectal cancers account for a substantial proportion of cancer-related mortality among American women; in particular, breast cancer represents the most frequently diagnosed malignancy, followed by colorectal and cervical cancers (Centers for Disease Control and Prevention (CDC), 2012). Several cancers affecting women have effective population-based screening strategies that can reduce morbidity and mortality through early detection and timely treatment (Centers for Disease Control and Prevention (CDC), 2012). In the United States, clinical guidelines recommend routine screening for breast, cervical, and colorectal cancers among eligible women; however, screening uptake remains suboptimal (Kenealy and Lochner, 2025; Centers for Disease Control and Prevention (CDC), 2012). National survey data consistently show that women with lower socioeconomic status, limited access to healthcare, and fewer preventive health behaviors are significantly less likely to be up to date with recommended screening, despite being at higher risk of adverse outcomes (Gorina and Elgaddal, 2021). Evidence indicates that regular screening substantially reduces cancer-related mortality. Modeling studies suggest that increasing screening uptake for breast, cervical, and colorectal cancers to recommended levels could prevent tens of thousands of premature deaths over the lifetime of eligible cohorts (Sharma et al., 2020). Nonetheless, even in high-income countries with established screening programs and widespread insurance coverage, participation gaps persist, underscoring the need for additional, scalable strategies to improve adherence to preventive services among women, particularly those facing structural, social, and economic barriers (Gorina and Elgaddal, 2021; Sharma et al., 2020). This persistent gap between evidence and uptake has prompted growing interest in strategies aimed at supporting individual decision-making and facilitating follow-through with recommended preventive behaviors.

Economic incentives are factors that encourage individuals to take specific actions. Various classifications of economic, monetary or financial incentives exist (Finkelstein et al., 2008; Ferris et al., 2019; Koch et al., 2024; Giles et al., 2016), such as monetary compensation, financial reimbursement, economic advantages, lottery winnings, vouchers, subscriptions, and discounts. The effectiveness of financial incentives differs among demographic groups and health issues.

From a behavioural economics perspective, financial incentives operate as nudges that counter present-bias, a cognitive tendency that leads individuals to prioritize immediate costs over future health benefits (Vlaev et al., 2019). By providing an immediate and tangible reward, incentives increase the short-term value of screening and help bridge the intention-action gap commonly observed in preventive behaviors (Kullgren et al., 2016). When benefits become salient, timely and easy to access, individuals are more likely to complete the required action, whereas delayed or abstract rewards are less motivating (Volpp et al., 2011).

In this framework, incentives do not act merely as compensation but as motivational levers that alter cost–benefit evaluations, reduce procrastination and support follow-through at the point of decision-making. Monetary rewards may enhance perceived relevance and facilitate adherence among individuals facing organizational, informational or emotional barriers (John et al., 2022). Evidence from preventive health programme shows that small but immediate incentives can increase adherence to actions such as vaccination, smoking cessation and weight-loss participation, particularly when paired with reminders or simplified pathways (Jecker, 2022; Cahill et al., 2015; Finkelstein et al., 2016). Notably, behavioural science suggests that intention-based interventions and nudges may be particularly effective among women, who tend to show higher engagement in preventive health behaviors and greater responsiveness to motivational cues (Carpenter, 2010).

A variety of measures have been tested to improve cancer screening participation: letters and telephone communications with physicians (Lantz et al., 1995), text messages (Hirst et al., 2017), videos on social media (Ruco et al., 2021) or television (Howe et al., 2002) and educational initiatives (Kim et al., 2022; Fung et al., 2021). This highlights the importance of understanding the broader implications of employing economic incentives to increase cancer screening adherence.

While economic and financial incentives targeting healthcare professionals have been extensively studied (Mauro et al., 2019; Pendrith et al., 2016), less evidence addresses incentives directed at eligible population. Participation in cervical, breast and colorectal cancer screening requires proactive engagement, but studies consistently report that organizational, educational and socioeconomic factors may limit women's attendance (Bongaerts et al., 2023; Mottram et al., 2021; Kotzur et al., 2022).

Therefore, evidence on how financial incentives influence screening adherence among women remains limited, as existing studies predominantly include mixed-gender populations and rarely examine women-specific outcomes across multiple screening programmes. This systematic review and meta-analysis aim to synthesize available studies across cervical, breast, and colorectal cancer screening programme to address this gap.

## 2 Methods

### 2.1 Data sources and search strategy

A protocol was developed for this systematic review and meta-analysis and registered online on PROSPERO (CRD42025640357), with minor procedural refinements detailed below.

We adopted the methodology of systematic reviews of effectiveness recommended by the Joanna Briggs Institute (Aromataris et al., 2024). Finally, our study was reported according to the Preferred Reporting Items for Systematic Reviews and Meta-Analysis (PRISMA) checklist (Page et al., 2021).

A research librarian from the University was consulted at the start of the search strategy to generate search strings (Supplementary, eTable 3). We consulted two main scientific databases, MEDLINE and CINAHL. Compared to the PROSPERO protocol, the Cochrane Library was additionally searched to maximize retrieval of randomized evidence. No other deviations from the protocol occurred.

### 2.2 Eligibility criteria and study selection

This review included quantitative studies evaluating the effect of economic, monetary or financial incentives on women eligible for cervical, breast or colorectal cancer screening (Supplementary, eTable 1). Eligible study designs were randomized controlled trials (RCTs) and quasi-experimental/observational studies with a comparison group. Comparators were defined as no incentive, usual care or standard invitation without financial benefit. Study protocols, grey literature, reviews and studies focused on cancer follow-up care were excluded. Only interventions offered within organized screening programme or opportunistic settings were considered. For the purposes of this review, organized screening programmes were defined as population-based programmes with systematic invitation and predefined screening protocols, whereas opportunistic screening referred to screening offered during routine healthcare encounters in the absence of a centralized invitation system.

All included studies recruited individuals designated as women in their original protocols. This classification was based on the existence of pertinent anatomy for screening objectives (e.g., cervix for cervical screening, breast tissue for mammography), regardless of gender identification. The terms women or female in this review are employed to align with the terminology utilized in the source research, rather than the participants' self-identified gender. To guarantee inclusivity, the research included persons assigned female at birth, trans men who have not undergone hysterectomy, individuals with breast tissue, trans women undergoing hormone therapy, and trans men without mastectomy. The PROSPERO protocol defined the population as women aged 25–69 years, to align with World Health Organization's cervical cancer screening recommendations. In the present review, the upper age threshold was extended to 75 years to align with international breast and colorectal screening recommendations in high-income countries, without altering the core eligibility framework. When reporting screening timeframes, the defined screening periods refer to the time window within which participants were required to complete the screening test to be eligible for the incentive, as specified in each primary study. These periods therefore reflect the conditional timing associated with incentive provision, rather than the recommended clinical screening interval.

Studies were eligible if they recruited women and reported outcomes relevant to cancer screening behaviors. The inclusion criteria did not require studies to be exclusively conducted in female populations. Accordingly, mixed-gender studies were included when screening outcomes for women could be extracted or were applicable to the target behavior.

When sex-disaggregated data were not available, results were analyzed as reported, without deriving additional sex-stratified estimates. Eligibility criteria were defined a priori according to the PICO framework (see eTable 2, Supplementary Material) and specified in the PROSPERO-registered protocol.

In the initial step of the selection process, all primary studies were included based on their titles and abstracts; in the second step, the full texts were evaluated. During both phases, the studies were independently selected by two reviewers, with the option available to include a third reviewer in cases of disagreement. Researchers were blinded to each other's decisions. The evaluation was performed via CADIMA software version 2.2.4.2.

### 2.3 Data extraction and quality assessment

Two reviewers independently extracted data. We gathered the following information: study ID and design, country and setting, eligibility criteria, number of women randomized/analyzed per group, intervention specifics and characteristics, control comparator, follow-up period, and main outcomes. Any discrepancies in data extraction were resolved through discussion and consensus, with consultation of a third reviewer when needed.

For studies including mixed-gender populations, female-specific outcomes were extracted when reported. When only aggregate data were available, results were analyzed as reported without deriving sex-stratified estimates.

The risk of bias was independently assessed by two reviewers via the Cochrane Risk of Bias 2 (RoB 2) tool for randomized trials and the Risk of Bias in Nonrandomized Studies of Interventions (ROBINS-I) tool for observational studies. Discrepancies were resolved by discussion or consultation with a third reviewer. Both instruments evaluate bias across key methodological domains, as described in the original frameworks.

Screening adherence, defined as the proportion of participants completing cancer screening within the follow-up period specified by each study, was selected as the primary outcome. No secondary outcomes were assessed.

### 2.4 Data synthesis and Meta-analysis

Interventions were grouped for synthesis: monetary incentives by value (e.g., $5, $10) and screening deadlines by timeframe (e.g., within 1 week or 1 year). For each study comparison, events and totals by arm were extracted, and risk ratios (RRs) were calculated via the Mantel-Haenszel method on a logarithmic scale, with a 0.5 continuity correction for zero counts. The meta-analysis included only randomized controlled trials to avoid bias from mixed designs (Bröckelmann et al., 2022). Owing to anticipated heterogeneity, the primary analysis used a random-effects model with restricted maximum likelihood (REML) estimation. In addition to 95% CIs, prediction intervals were reported to reflect the expected effects in new studies. Heterogeneity was assessed using Cochran's Q statistic, the I^2^ statistic, and τ (Centers for Disease Control and Prevention (CDC), 2012).

When studies included multiple intervention arms, comparisons were included according to the analytic grouping strategy to avoid double counting. Table 1 reports intervention arms as described in the original studies, whereas Fig. 2, Fig. 3 reflect the pooled comparisons included in the meta-analysis. Where relevant, the control group was split evenly across comparisons following standard Cochrane Review recommendations.

**Table 1:** Characteristics of quantitative studies evaluating financial incentives to improve cervical, breast, and colorectal cancer screening participation among women in the United States and India, published between 2005 and 2022. Overview of included randomized and observational studies.

Study ID and Authors	Country and Time Published	Interventions	Control group	Time window⁎	Screening Type	Study Design
Choudhury, H. K.; Borah, R. K.	India, 2022	Monetary incentive of Rs. 150 ($1.71)⁎⁎	No monetary incentive	Within the scheduled screening period (3 weeks)	Cervical Cancer (HPV⁎⁎⁎ test)	RCT⁎⁎⁎⁎
Gupta, S.; Miller, S.; Koch, M. et al.	United States, 2016	$5 or $10 of incentive	No monetary incentive	Within the recommended screening period (1 year)	Colorectal Cancer (FIT/FOBT⁎⁎⁎⁎⁎ completion)	RCT
Lieberman, A.; Gneezy, A.; Berry, E. et al.	United States, 2021	Two conditional incentive schedules: Arm 1: $10 for completion within 1 week or $5 within 3 weeks Arm 2: $20 for completion within 1 week or $10 within 3 weeks	No incentive and no deadline for completion	Within the study follow-up period (1 week and 3 weeks)	Colorectal Cancer (FIT return)	RCT
Mehta, S. J.; Pepe, R. S.; Gabler, N. B. et al.	United States, 2019	$10 store gift card along with their FIT within 2 months. Lottery with a 1-in-10 chance of receiving a $100 gift card.	No incentive	Within the study follow-up period (2 months)	Colorectal Cancer (FIT/FOBT completion)	RCT
Slater, J. S.; Henly, G. A.; Ha, C. N. et al.	United States, 2005	$10 monetary incentive	No incentive, no e-mail of invitation	Within the study follow-up period (1 year)	Breast Cancer (Mammography)	RCT
Slater, J. S.; Parks, M. J.; Malone, M. E. et al.	United States, 2017	$25 monetary incentive	No incentive, no e-mail of invitation	Within the study follow-up period (1 year)	Breast Cancer (Mammography)	RCT
Slater, J. S.; Parks, M. J.; Nelson, C. L.; Hughes, K. D.	United States, 2018	$20 monetary incentive	No incentive, no e-mail of invitation	Within the study follow-up period (3 months)	Breast cancer (mammography) and colorectal cancer (colonoscopy)	RCT
Mehta, SJ; Reitz, C; Niewood, T. et al.	United States, 2021	$10 conditional incentive (risk assessment) $25 unconditional incentive (colonoscopy)	No incentive and no completion of risk assessment	Within the study follow-up period (1 year)	Colorectal cancer (risk assessment and colonoscopy)	RCT
Mehta, SJ; Oyalowo, A; Reitz, C. et al.	United States, 2020	1-in-5 chance of winning a $100 gift card after FIT completion.	No incentive, only SMS⁎⁎⁎⁎⁎⁎ of invitation	Within the study follow-up period (3 months)	Colorectal Cancer (FIT/FOBT completion)	RCT
Litaker, J. R.; Tamez, N.; Durkalski, W.; Taylor, R.	United States, 2021	Outreach and education on no-cost screening mammography plus a $50 grocery gift card conditional on completion of screening within a defined time window.	Historical comparison period without outreach, education, or incentive	Within the study follow-up period	Breast Cancer (Mammography)	Cohort Study

⁎ Time windows reflect the period within which screening completion was required to be eligible for the incentive, as defined in each primary study or, when no explicit deadline was specified, correspond to the study follow-up period;

⁎⁎ Monetary incentive of 150 Indian Rupees (INR), equivalent to approximately USD $1.71;

⁎⁎⁎ HPV: Human Papillomavirus;

⁎⁎⁎⁎ RCT: Randomized Controlled Trial;

⁎⁎⁎⁎⁎ FIT: Fecal Immunochemical Test and FOBT: Fecal Occult Blood Test;

⁎⁎⁎⁎⁎⁎ SMS: short message service (text message).

Prespecified subgroup analyses focused on follow-up duration, given its theoretical relevance for interpreting incentive effects and its role as a major source of between-study heterogeneity. Follow-up windows were categorized as ≤1 month, 2–6 months, and approximately 1 year.

Data were analyzed using R version 4.2.1 (meta and metafor packages).

We assessed the certainty of evidence via the GRADE approach, rating evidence as high, moderate, low, or very low. Ratings were downgraded by 1 or 2 levels for serious concerns (e.g., high risk of bias in key trials, substantial heterogeneity [I^2^ ≥ 60%], or broad prediction intervals). Publication bias was judged based on trial registration, adherence to CONSORT (CONsolidated Standards of Reporting Trials) reporting, and journal characteristics. (peer-reviewed, indexed, open-access policy).

## 3 Results

**Fig. 1:**
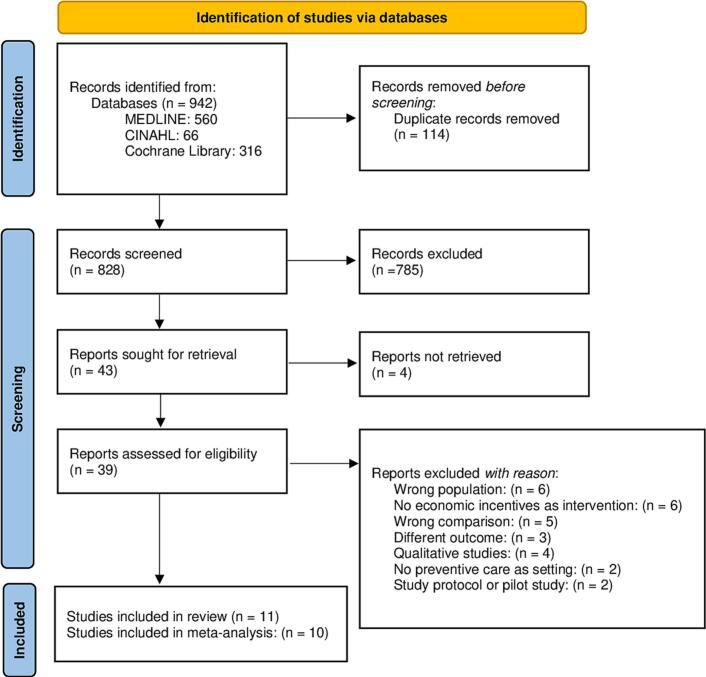
PRISMA flow diagram of studies evaluating financial incentives to improve cervical, breast, and colorectal cancer screening participation among women in the United States and India, published between 2005 and 2022. Flowchart illustrating identification, screening, eligibility assessment and final inclusion of studies evaluating financial incentives to increase cancer screening participation. Records were retrieved through database searching and other sources, screened by title/abstract and full text, and studies meeting eligibility criteria were included.

Our search yielded a total of 942 results. Among them, 39 studies were considered eligible, and 11 were included in the review: 10 randomized controlled trials and 1 cohort study. Fig. 1 presents the PRISMA flow diagram, illustrating the research selection procedure and reasons for exclusions. All the studies were published in English.

The eleven studies included 20,152 women: six were conducted in socioeconomically disadvantaged communities [S1-S6] and four additional studies addressed general populations [S7-S11].

Table 1 summarizes the characteristics of the included RCTs [S1-S10] and the cohort study [S11]. Interventions spanned different formats: monetary incentives alone [S3, S6]; incentives tied to deadlines [S1, S9]; incentives such as gift cards [S10, S11]; financial rewards combined with mailed educational materials [S2, S7, S8]; incentives linked to cancer risk assessment [S11]; and text messaging paired with incentives [S6].

All trials compared financial incentives against standard care (e.g., direct mail, text messages, or screening invitations with colorectal test kits). The median follow-up was 3 months (IQR, 1–6).

Except for one study [S6] conducted in India, a lower-middle-income country, all the studies were U.S.-based. Funding primarily came from national or university research centers, with two exceptions: one supported by the 10.13039/100000030Centers for Disease Control and Prevention (CDC) [S8] and another by a private insurer [S11].

The methodological quality was generally moderate. Most RCTs clearly reported eligibility, interventions, and outcomes, but participant/professional blinding was rarely feasible, and assessor blinding was often insufficiently described. Follow-up was adequate with registry-based outcomes, although reporting of withdrawals was limited. Statistical analyses were mostly appropriate, with occasional issues in baseline comparability.

The nonrandomized cohort study met several checklist criteria (aims, setting, and exposure measurement) but showed weaknesses in terms of confounding control, follow-up completeness, and potential outcome bias. eFigure 1 and eFigure 2 (Supplementary) present the risk of bias for RCTs (RoB 2) and the cohort study (ROBINS-I). Overall, the quality of the RCTs was suboptimal: none were at low overall risk, 5 were at high risk, and 5 showed some concerns. Randomization was frequently unclear due to poor reporting of sequences or concealment. The most critical issue was deviations from intervention, given the lack of blinding in incentive-based designs, with several studies at high risk and limited ITT analyses. The risk of bias from missing data was generally low. The outcome measurement was judged to be of low risk or with some concerns given the reliance on objective registry data. Only 1 study was assessed as having a high risk for outcome misclassification. Selective reporting was mostly low risk.

For the cohort study [S11], the risk of confounding and deviations was serious, selection was moderate, and outcome measurement was serious, with other domains being low. Overall quality was judged as serious.

Ten randomized controlled trials and one cohort study met the eligibility criteria (Supplementary, eTable 4). The cohort study was not pooled with the randomized trials and is reported separately.

**Fig. 2:**
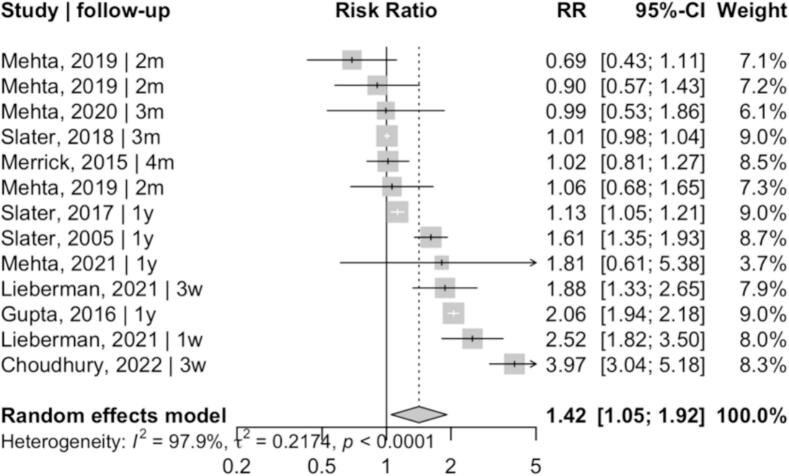
Forest plot of randomized controlled trials evaluating financial incentives to improve cervical, breast, and colorectal cancer screening participation among women in the United States and India, published between 2005 and 2022. Risk ratios (RR) with 95% confidence intervals are presented for all included randomized controlled trials, with studies labeled according to follow-up timing. The pooled estimate was calculated using a random-effects model (REML with Hartung-Knapp adjustment). Substantial heterogeneity reflects differences in intervention formats, screening contexts, and outcome assessment windows across studies.

Across individual studies, most effect estimates favored financial incentives, although effect size and confidence intervals varied markedly. Using a random-effects model (REML with Hartung-Knapp adjustment), pooled results indicated increased screening participation associated with financial incentives - RR 1.42 (95% CI 1.05, 1.92); I^2^ = 98.30% (Fig. 2). The cohort study [S11], which was not pooled with randomized trials, reported a positive association between incentives and screening adherence - RR 2.03 (95% CI 1.62, 2.54). However, this finding should be interpreted cautiously due to serious risk of confounding and was not included in the meta-analysis.

**Fig. 3:**
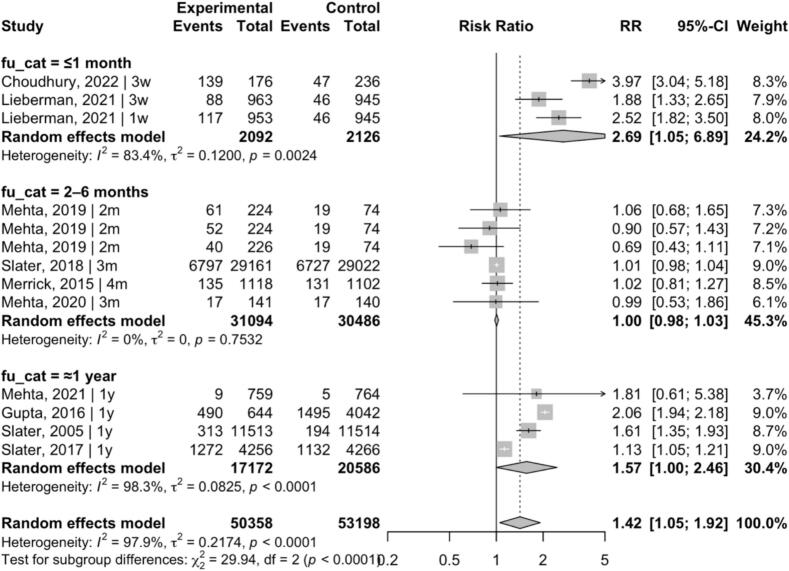
Random-effects meta-analysis stratified by follow-up timing of randomized controlled trials evaluating financial incentives to improve cervical, breast, and colorectal cancer screening participation among women in the United States and India, published between 2005 and 2022. Random-effects meta-analysis of randomized controlled trials evaluating financial incentives for breast, cervical, and colorectal cancer screening among women, stratified by follow-up window (≤1 month, 2–6 months, and approximately 1 year). Differences across strata should be interpreted cautiously, as follow-up timing overlapped with the type of incentive used in several trials (particularly lottery-based incentives), limiting causal interpretation of subgroup differences.

Subgroup analyses were conducted by follow-up duration to explore whether the timing of outcome assessment contributed to the observed heterogeneity (Fig. 3). Effect estimates varied across follow-up intervals, with larger point estimates observed in studies assessing screening uptake within ≤1 month - RR 2.69 (95% CI 1.05, 6.89) - estimates at 2–6 months were close to null - RR 1.00 (95% CI 0.98–1.03) - but effects were statistically detectable at approximately 1 year - RR 1.57 (95% CI 1.00, 2.46). However, these differences likely reflect variation in target behaviors and intervention characteristics rather than distinct effects of follow-up timing. Between-subgroup differences were statistically significant (χ^2^ = 29.94, df = 2, *p* < 0.01) but given the small number of studies and residual heterogeneity within subgroups, these findings remain exploratory.

Certainty of evidence was judged to be low for the overall pooled effect, downgraded for very high heterogeneity (I^2^ = 98.3%). Certainty was moderate for studies assessing outcomes within ≤1 month, low at 2–6 months, and moderate at approximately 1 year. Evidence from the single cohort study contributed very low-certainty evidence due to serious risk of bias.

## 4 Discussion

Preventive care remains a central yet unresolved challenge in women's health, particularly for conditions in which effective interventions exist but are not consistently utilized. Persistent gaps in participation in cancer screening highlight the disconnect between evidence-based recommendations and real-world adherence. Addressing this implementation gap is essential to reducing avoidable morbidity and advancing equity in women's health.

Across studies, moderate-value incentives (approximately $10–25) were most frequently associated with positive effects; although, formal comparisons by incentive amount were limited by heterogeneity and imprecision. Effect estimates also varied across follow-up windows; however, these differences do not support a single optimal follow-up duration and are strongly context-dependent, as follow-up timing is closely linked to the type and burden of the target screening behavior and to intervention characteristics. The observed heterogeneity likely reflects the diversity of target behaviors, intervention designs, and contextual factors across studies, rather than inconsistency in the effectiveness of financial incentives per se. This reinforces that heterogeneity is an inherent characteristic of incentive-based interventions, not merely a statistical artifact.

Accordingly, the pooled estimate should primarily be interpreted as reflecting the overall direction of the association rather than a precise estimate of effect magnitude. This interpretation is consistent with the high heterogeneity and the diversity of cancer screened, intervention formats and screening contexts represented in the included trials.

Follow-up timing should be interpreted in relation to the target screening behavior. The time required to complete a screening test varies substantially depending on its complexity, accessibility, and perceived burden. For example, completing a brief assessment may reasonably occur within a short time frame, whereas procedures such as colonoscopy require longer and more flexible time windows. Accordingly, our findings should not be interpreted as identifying an optimal follow-up duration. Rather, they suggest that financial incentives may be associated with screening uptake across a range of time windows, depending on the context and characteristics of the target behavior.

Despite overall favorable trends, the certainty of evidence remains limited by methodological variability, heterogeneity and short follow-up. Several randomized trials suggest that economic incentives may promote screening participation [S2, S6-S8], yet confidence is tempered by high between-study inconsistency and risk of bias. Differences in effect estimates across follow-up windows were observed, with larger effects when screening completion was assessed shortly after incentive delivery. However, follow-up timing was strongly correlated with the type of incentive used in several trials, particularly lottery-based incentives concentrated in the 2–6 month window. Therefore, follow-up duration cannot be interpreted as an independent explanation of heterogeneity.

Residual variability likely reflects unmeasured moderators – baseline uptake, screening infrastructure, recruitment strategies, and contextual differences - none of which were consistently documented. Incentive delivery also varied (cash transfers, gift cards, conditional deadlines, mailed materials), preventing firm conclusions on format effectiveness. Effect estimates appeared smaller on average at longer follow-up assessments, although these differences should be interpreted cautiously given variability in study characteristics. In addition, several trials used lottery-based incentives rather than fixed payments. Lottery incentives rely on behavioural mechanisms such as uncertainty and probability weighting, which differ from guaranteed rewards. These differences in incentive format may represent an additional source of variability in observed effects across studies.

Most studies were conducted in the US and among insured populations, limiting generalizability to publicly funded or low-resource programme. Participants were often already engaged in care, which may inflate estimated adherence. Ethical implications, sustainability of incentive-based policies, and potential unintended inequities remain largely unexplored. Individual factors – including socioeconomic disadvantage, prior screening experience and health literacy (Hassine et al., 2022) - may influence responsiveness, but evidence is still fragmentary. Health system variables such as program organization, reimbursement mechanisms, cost of implementation and long-term scaling are also insufficiently investigated. To better interpret these results, contextual factors across countries, behavioural mechanisms, and population differences must also be considered.

The only available study evaluating incentives for HPV screening [S6] suggested potential effectiveness; however, interpretation is limited by the specific sociocultural and economic context in which the study was conducted. HPV screening is influenced by stigma and sociocultural norms (Waller et al., 2007), and incentives may mitigate perceived barriers by increasing immediate motivation. Yet, this evidence derives from a trial in India, where 150 INR (≈1.6 EUR / < 2 USD). Monetary value is therefore not comparable across settings or decades - a concern also highlighted by reviewers - and incentives used historically would require inflation adjustment when interpreted in present-day equivalence. Consequently, effectiveness observed in low-income contexts may not translate directly to high-income countries, and cross-country applicability remains uncertain.

Most remaining studies were conducted in the United States, within an insurance-based health system, which limits transferability to countries with universal coverage. Some interventions among privately insured groups [S10, S11] were ineffective, possibly reflecting higher baseline access to healthcare or preference for non-financial person-centred benefits (e.g., gym passes, museum tickets). Conversely, $20 incentives improved uptake among Medicaid-insured low-income women [S2], whereas $5–10 incentives showed no effect in highly vulnerable groups [S3]. This suggests that incentive impact may vary by socioeconomic need and perceived value, supporting the hypothesis that context-dependent calibration rather than incentive size alone determines effectiveness.

A behavioural perspective is also relevant. The effectiveness of financial incentives is likely shaped by the effort required to complete different screening procedures, including preparation, time commitment, and logistical demands. Monetary incentive values in the included studies were generally modest, with the highest value being $25. Consequently, the available evidence does not allow conclusions about whether larger financial incentives might produce stronger effects for screening procedures requiring greater preparation, time commitment, or logistical demands. Mechanisms that facilitate action – such as deadlines [S1] or sequential reminders [S3, S4] - may still support follow-through in line with behavioural-economics theory suggesting that immediacy and friction-reduction enhance behavior change [S7]. This reinforces the idea that incentive design should be tailored to the behavioural and practical demands of specific screening modalities, and that intervention design may influence how financial incentives translate into behavior change. Accordingly, future interventions should consider combining incentives with outreach, navigation support, or personalized communication.

This review extends previous work by analyzing incentives across three major screening programmes rather than colorectal screening alone. Consistent with prior findings (Facciorusso et al., 2021), incentives produced modest absolute gains (∼93 additional women screened per 1000). Moderate-value incentives (approximately $10–25) were most evaluated across studies, although formal comparisons across incentive magnitudes were limited by low number of studies, heterogeneity and variation in incentive formats. Contrary to prior conclusions suggesting limited effectiveness of financial incentives in underserved populations (Facciorusso et al., 2021), the present review did not find systematically smaller effects in socioeconomically disadvantaged women, although confidence intervals were wide. Evidence on incentive schedule suggests that smaller sequential payments may outperform single transfers (Nisa et al., 2019). Observations align with UK studies using £10–20 vouchers (Wilding et al., 2022) and research showing increased survey participation with $10 unconditional incentives in families of pediatric cancer survivors.

This review has several limitations. First, although PROSPERO registration preceded data extraction, operational thresholds for incentive categories ($ < 5, $10–25, $ > 25) were defined post-hoc during synthesis, reflecting the absence of standardized cut-offs in the literature. Subgroup analyses were exploratory and underpowered, and the decision not to perform meta-regression was based on the small number of eligible trials to avoid overfitting.

Second, although search strategies were comprehensive across MEDLINE, CINAHL and Cochrane, we did not search grey literature or trial registries beyond PROSPERO, which may have excluded unpublished studies and contributed to small-study effects. Third, monetary values were extracted as reported, without adjustment for inflation or purchasing power parity across decades. Consequently, a $10 incentive in 1995 does not equate to $10 in 2025, potentially biasing comparisons. Fourth, screening outcomes were based on completion within study-defined windows, which varied between trials, limiting temporal comparability. Although subgroup analyses reduced heterogeneity partially, residual inconsistency remained substantial, and unmeasured moderators – such as baseline screening rates, outreach strategy intensity, cultural norms, and perceived value of incentives - may have contributed.

Finally, some included studies did not report sex-specific outcomes. In these cases, aggregate data were used, which may limit the precision of inferences specific to women.

Despite these limitations, strengths include prospective protocol registration, duplicate screening/extraction, structured risk-of-bias evaluation, and the use of random-effects models with prediction intervals to support external applicability.

Future trials should harmonize outcome timings, adjust incentive values to contemporary currency equivalence, and incorporate cost-effectiveness and acceptability measures. Studies beyond the United States, particularly within publicly funded European systems, are necessary to evaluate scalability. Future research should also examine the role of financial incentives among women at elevated clinical risk for cancer, while distinguishing this group from populations facing structural or socioeconomic barriers to screening.

Policymakers considering incentives should integrate them within broader outreach frameworks and monitor equity impacts. Long-term follow-up is required to determine whether incentives influence sustained adherence across screening rounds.

## 5 Conclusion

Financial incentives show potential as a pragmatic tool to improve cancer screening adherence among women. However, the high heterogeneity, low certainty of evidence, and methodological limitations limit the precision and generalizability of these estimates. Further well-designed, equity-focused trials are needed to determine which incentive models are most effective across different screening contexts and time windows.

## Patients consent statement

Not applicable.

## Data availability and materials statement

All materials supporting the findings of this review are included in the published article and its supplements. Additional data not publicly posted can be obtained from the corresponding author upon reasonable request.

## CRediT authorship contribution statement

**Cristina Lumia:** Writing – review & editing, Writing – original draft, Software, Methodology, Investigation, Formal analysis, Data curation, Conceptualization. **Antonella Nespoli:** Writing – original draft, Validation, Supervision, Investigation, Data curation. **Davide Luigi Lino Ausili:** Validation, Supervision. **Simona Fumagalli:** Supervision, Software, Methodology, Conceptualization. **Alexandre Dumont:** Writing – review & editing, Writing – original draft, Validation, Supervision, Methodology, Formal analysis, Data curation.

## Ethical approval statement

Not applicable.

## Funding statement

This research received no specific grant from any funding agency in the public, commercial, or not-for-profit sectors.

## Declaration of Competing Interest

The authors declare that they have no known competing financial interests or personal relationships that could have appeared to influence the work reported in this paper.

## Data Availability

Data will be made available on request.

## Appendix A Supplementary data

Additional methodological details and supporting materials for the systematic review and meta-analysis, including screening eligibility criteria, PICO-based eligibility criteria, database search strategy, risk of bias assessments, and references of included studies.
