# Supplementary material for: Financial incentives for women's cervical, breast and colorectal cancer screening adherence: systematic review and meta-analysis
Source: Prev Med Rep. 2026 May 25;67:103507. doi: 10.1016/j.pmedr.2026.103507 (PMC13241874; doi:10.1016/j.pmedr.2026.103507)
Supplement: Supplementary file 1 — Additional methodological details and supporting materials for the systematic review and meta-analysis, including screening eligibility criteria, PICO-based eligibility criteria, database search strategy, risk of bias assessments, and references of included studies. [file mmc1.docx]

**Financial incentives for women's cervical, breast and colorectal cancer screening adherence: systematic review and meta-analysis**

**eTable 1. Eligibility criteria for screening tests***

Summary of eligibility criteria for cervical, breast and colorectal cancer screening programme.

| **Country** | **Cervical cancer age** | **Breast cancer**  **age** | **Colorectal**  **age** |
| --- | --- | --- | --- |
| **European Union Recommendation** | 30–65 | 45–74 | 50–74 |
| **Switzerland** | Opportunistic: 21-70  Preference: 30–70 | Typically: 50–69 | Typically: 50–69 |
| **Italy** | 25–64 (HPV 30–64) | 50–69 | 50–69 (some regions until 74) |
| **United Kingdom** | 25–64 | 50–71 | 50–74 |
| **USA** | 21–65 | 40–74 | 45–75 |
| **Canada** | 25–69 | 50–74 | 50–74 |
| **Australia** | 25–74 | 50–74 | 50–74 |
| **France** | 25–65 | 50–74 | 50–74 |
| **Germany** | 20–65 | 50–69 | Da 50+ |
| **Netherlands** | 30–60 | 50–75 | 55–75 |

* Official Journal of the European Union; Cancer Screening Committee; Ministero della Salute; National Health Service (NHS); American College of Obstetricians and Gynecologists (ACOG); Institut National du Cancer.

**eTable 2. Eligibility criteria according to PICO**

Eligibility criteria used to select studies, organized according to the PICO framework (Population, Intervention, Comparator, Outcomes).

| **POPULATION** | Women ≥25 years eligible for breast, cervical or colorectal cancer screening. |
| --- | --- |
| **INTERVENTION** | Financial/economic incentives (cash, vouchers, reimbursements, gift cards, lotteries or equivalent benefits). |
| **COMPARATOR** | No incentive/usual invitation to screening. |
| **OUTCOME** | Screening adherence/completion. |

**eTable 3. Search strategy**

Search strings and parameters applied in database querying.

| Database | Search string |
| --- | --- |
| MEDLINE  (19/02/2025) | ((economic OR financial OR monetary) incentive* OR Reimbursement, Incentive OR Economics, Behavioral OR Reward) (Early Detection of Cancer OR Mass Screening) (Female OR Women OR females OR woman OR Transgender Persons OR Transsexualism)  (“Financial Incentives"[Mesh]  OR incentive*[tiab]  OR "financial incentive*"[tiab]  OR "economic incentive*"[tiab]  OR "monetary incentive*"[tiab]  OR "behavioural economics"[tiab]  OR reimbursement*[tiab]  OR "cash" OR "voucher*" OR "gift card*" OR "reward*”)  AND  (“Early Detection of Cancer"[Mesh]  OR "Mass Screening"[Mesh]  OR "Health Screening"[tiab]  OR "Cancer Screening"[tiab]  OR screening[tiab]  OR uptake[tiab]  OR participation[tiab]  OR adherence[tiab]  OR compliance[tiab]  OR "screening invitation*"[tiab]  OR "screening reminder*"[tiab])  AND  (“Breast Neoplasms/diagnosis"[Mesh]  OR "Mammography"[Mesh]  OR breast screening[tiab]  OR mammography[tiab]  OR"Uterine Cervical Neoplasms/diagnosis"[Mesh]  OR "Papanicolaou Test"[Mesh]  OR cervical screening[tiab]  OR Pap smear[tiab]  OR HPV test*[tiab]  OR "Colorectal Neoplasms/diagnosis"[Mesh]  OR "Colonoscopy"[Mesh]  OR "Fecal Occult Blood Test"[Mesh]  OR colorectal screening[tiab]  OR colonoscopy[tiab]  OR FOBT [tiab]  OR FIT [tiab])  AND  (female*[tiab] OR women[tiab] OR woman[tiab]  OR "Female"[Mesh]  OR transgender*[tiab] OR "Transgender Persons"[Mesh]) |
| CINAHL  (20/02/2025) | (MM "Reimbursement, Incentive" OR ((economic OR financial OR monetary) incentive*) OR MM "Economic Factors" OR MM "Reward") AND (MM "Early Detection of Cancer" OR MM "Cancer Screening" OR MM "Health Screening+") (females OR woman OR MH "Female" OR MH "Women+" OR MH "Transgender Persons+" OR MH "Transsexual Persons")  (MH "Reimbursement, Incentive"  OR MH "Economic Factors"  OR MH "Behavioral Economics"  OR incentive*  OR "financial incentive*"  OR "economic incentive*"  OR "monetary incentive*"  OR voucher* OR payoff* OR reward*)  AND  (MH "Early Detection of Cancer"  OR MH "Mass Screening"  OR MH "Cancer Screening"  OR screening OR uptake OR adherence OR compliance  OR participation OR "screening invitation*" OR "screening reminder*”)  AND  (MH "Breast Neoplasms" OR MH "Mammography" OR breast screening OR mammography  OR MH "Uterine Cervical Neoplasms" OR MH "Papanicolaou Test" OR cervical screening OR Pap smear OR HPV test*  OR MH "Colorectal Neoplasms" OR MH "Colonoscopy" OR colorectal screening OR FOBT OR FIT)  AND  (MH "Female" OR MH "Women" OR females OR women OR woman  OR MH "Transgender Persons") |
| COCHRANE LIBRARY  (20/02/2025) | incentive* Screening (Female OR women OR Transgender OR Transsexualism)  (incentive* OR "financial incentive*" OR "economic incentive*" OR "monetary incentive*" OR reward* OR voucher*)  AND  (screening OR "cancer screening" OR uptake OR participation OR adherence OR compliance)  AND  (breast OR mammograph*  OR cervical OR "pap smear" OR "HPV test"  OR colorectal OR colonoscopy OR FOBT OR FIT)  AND  (women OR female*) |

**eFigure 1. Risk of bias for randomized controlled trials**

**
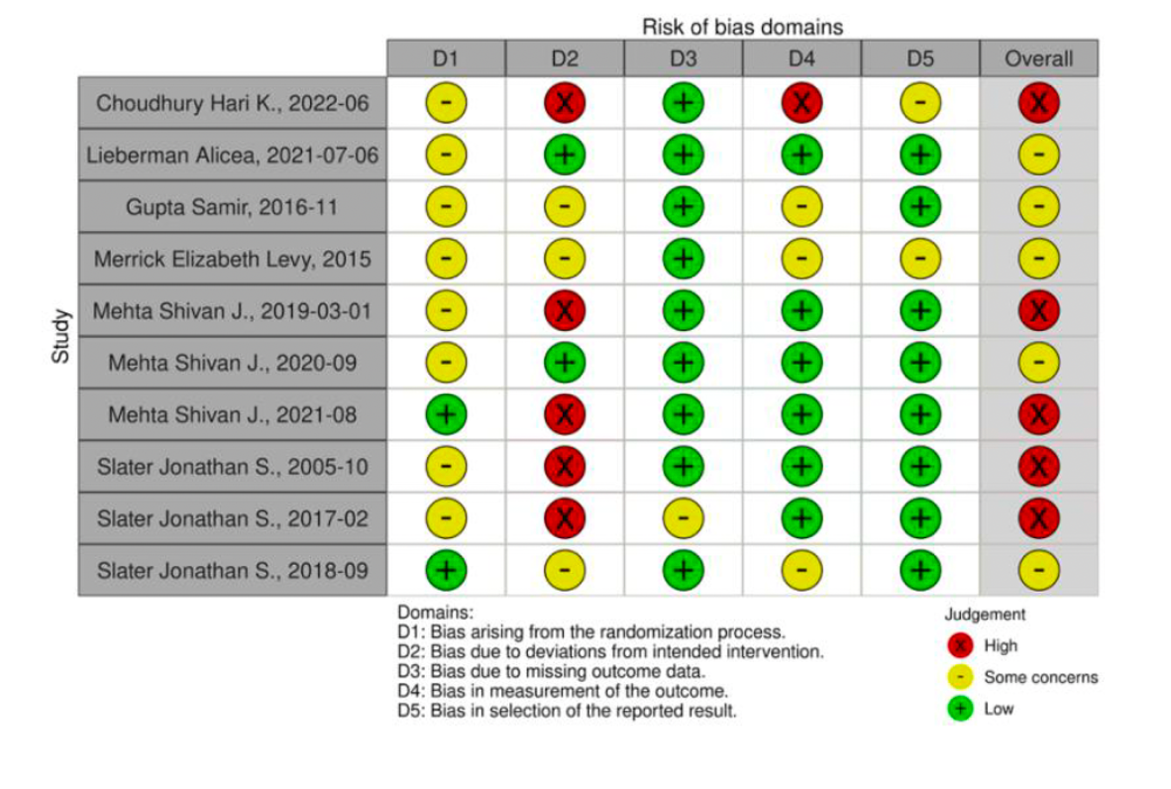
**

*Summary of RoB2 domain ratings and overall risk of bias judgement for included randomized controlled trials.*

**eFigure 2. Risk of bias for nonrandomized study**

**
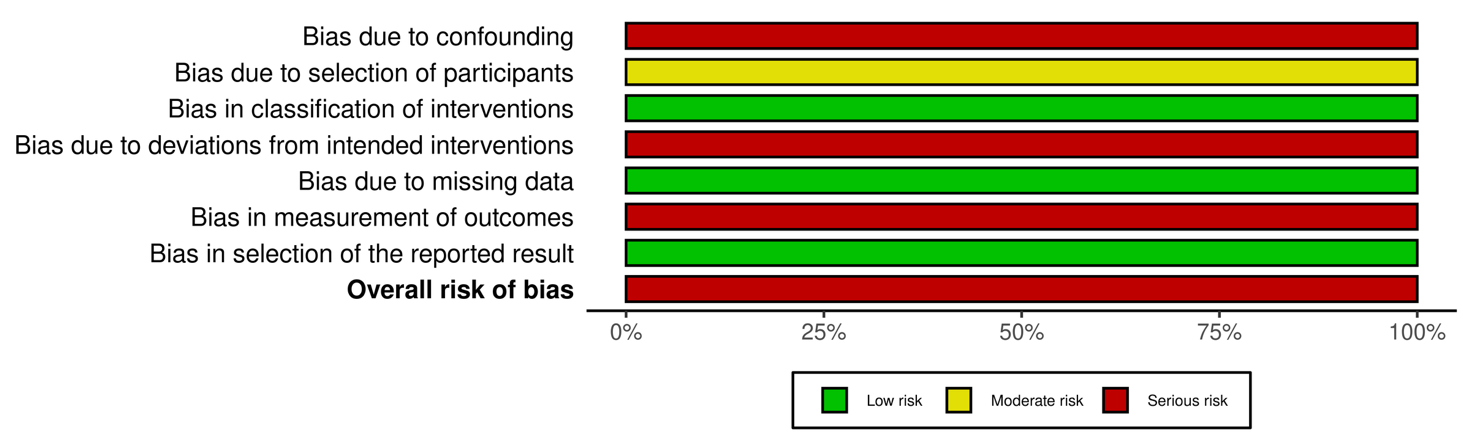
**

*Risk of bias assessment for non-randomized study using the ROBINS-I tool.*

**eReferences.**

List of included studies of systematic review and meta-analysis of financial incentives for women's cervical, breast and colorectal cancer screening adherence.

S1. Lieberman A, Gneezy A, Berry E, et al. The effect of deadlines on cancer screening completion: a randomized controlled trial. Sci Rep. 2021;11(1):13876. doi:10.1038/s41598-021-93334-1

S2. Slater JS, Parks MJ, Nelson CL, Hughes KD. The efficacy of direct mail, patient navigation, and incentives for increasing mammography and colonoscopy in the Medicaid population: a randomized controlled trial. Cancer Epidemiol Biomarkers Prev. 2018;27(9):1047-1056. doi:10.1158/1055-9965.EPI-18-0038

S3. Gupta S, Miller S, Koch M, et al. Financial incentives for promoting colorectal cancer screening: a randomized, comparative effectiveness trial. Am J Gastroenterol. 2016;111(11):1630-1636. doi:10.1038/ajg.2016.286

S4. Slater JS, Henly GA, Ha CN, et al. Effect of direct mail as a population-based strategy to increase mammography use among low-income underinsured women ages 40 to 64 years. Cancer Epidemiol Biomarkers Prev. 2005;14(10):2346-2352. doi:10.1158/1055-9965.EPI-05-0034

S5. Mehta SJ, Oyalowo A, Reitz C, et al. Text messaging and lottery incentive to improve colorectal cancer screening outreach at a community health center: a randomized controlled trial. Prev Med Rep. 2020;19:101114. doi:10.1016/j.pmedr.2020.101114

S6. Choudhury HK, Borah RK. Can financial incentives encourage women to participate in a cervical cancer screening programme? Evidence from a randomized controlled trial analysis. J Cancer Policy. 2022;32:100324. doi:10.1016/j.jcpo.2022.100324

S7. Mehta SJ, Reitz C, Niewood T, Volpp KG, Asch DA. Effect of behavioral economic incentives for colorectal cancer screening in a randomized trial. Clin Gastroenterol Hepatol. 2021;19(8):1635-1641.e1. doi:10.1016/j.cgh.2020.06.047

S8. Slater JS, Parks MJ, Malone ME, et al. Coupling financial incentives with direct mail in population-based practice. Health Educ Behav. 2017;44(1):165-174. doi:10.1177/1090198116646714

S9. Mehta SJ, Pepe RS, Gabler NB, et al. Effect of financial incentives on patient use of mailed colorectal cancer screening tests: a randomized clinical trial. JAMA Netw Open. 2019;2(3):e191156. doi:10.1001/jamanetworkopen.2019.1156. Erratum in: JAMA Netw Open. 2019;2(4):e193771. doi:10.1001/jamanetworkopen.2019.3771

S10. Merrick EL, Hodgkin D, Horgan CM, et al. Testing novel patient financial incentives to increase breast cancer screening. Am J Manag Care. 2015;21(11):771-779.

S11. Litaker JR, Tamez N, Durkalski W, Taylor R. A cue-to-action pilot project to increase screening mammography. Am J Manag Care. 2021;27(2):e48-e53. doi:10.37765/ajmc.2021.88589
